# Supplementary material for: Molecular subtypes of ALS are associated with differences in patient prognosis
Source: Nat Commun. 2023 Jan 6;14:95. doi: 10.1038/s41467-022-35494-w (PMC9822908; doi:10.1038/s41467-022-35494-w)
Supplement: Supplementary file 1 — Supplementary Information [file 41467_2022_35494_MOESM1_ESM.pdf]

Supplementary Information for

Molecular subtypes of ALS are associated with differences in patient prognosis

Jarrett Eshima, Samantha A. O'Connor, Ethan Marschall, NYGC ALS Consortium, Robert Bowser, Christopher L. Plaisier, Barbara S. Smith

Correspondence to: BarbaraSmith@asu.edu

This PDF file includes:

Supplementary Text
Figs. S1 to S16
Tables S1 and S2

Other Supplementary Materials for this manuscript include the following:

Supplementary Data 1-12

Supplementary Text

NYGC ALS Consortium Members and sites.

Hemali Phatnani[1], Justin Kwan[2], Dhruv Sareen[3,4,5], James R. Broach[6], Zachary Simmons[7], Ximena Arcila-Londono[8], Edward B. Lee[9], Vivianna M. Van Deerlin[9], Neil A. Shneider[10], Ernest Fraenkel[11], Lyle W. Ostrow[12], Frank Baas[13,14], Noah Zaitlen[15], James D. Berry[16,17], Andrea Malaspina[18,19,20], Pietro Fratta[21], Gregory A. Cox[22], Leslie M. Thompson[23,24,25], Steve Finkbeiner[26], Efthimios Dardiotis[27], Timothy M. Miller[28], Siddharthan Chandran[29,30], Suvankar Pal[29,30], Eran Hornstein[31], Daniel J. MacGowan[32], Terry Heiman-Patterson[33], Molly G. Hammell[34], Nikolaos A. Patsopoulos[35,36,37,38], Oleg Butovsky[39], Joshua Dubnau[40], Avindra Nath[41], Robert Bowser[42,43], Matthew Harms[44], Eleonora Aronica[45], Mary Poss[46], Jennifer Phillips-Cremins[47], John Crary[48,49], Nazem Atassi[50], Dale J. Lange[51], Darius J. Adams[52,53], Leonidas Stefanis[54,55], Marc Gotkine[56], Robert H. Baloh[4,57], Suma Babu[17], Towfique Raj[49,58,59], Sabrina Paganoni[60], Ophir Shalem[61,62], Colin Smith[63,64], Bin Zhang[59], University of Maryland Brain and Tissue Bank, NIH NeuroBioBank, Brent Harris[65,66], Iris Broce[67], Vivian Drory[68,69], John Ravits[70], Corey McMillan[71], Vilas Menon[72]

Affiliations:

[1]Center for Genomics of Neurodegenerative Disease (CGND), New York Genome Center, New York, NY, USA
[2]Department of Neurology, Lewis Katz School of Medicine, Temple University, Philadelphia, PA, USA
[3]Cedars-Sinai Department of Biomedical Sciences, Cedars-Sinai Medical Center, Los Angeles, CA, USA
[4]Board of Governors Regenerative Medicine Institute and Brain Program, Cedars-Sinai Medical Center, Los Angeles, CA, USA
[5]Department of Medicine, University of California, Los Angeles, CA, USA
[6]Department of Biochemistry and Molecular Biology, Penn State Institute for Personalized Medicine, The Pennsylvania State University, Hershey, PA, USA
[7]Department of Neurology, The Pennsylvania State University, Hershey, PA, USA
[8]Department of Neurology, Henry Ford Health System, Detroit, MI, USA
[9]Department of Pathology and Laboratory Medicine, Perelman School of Medicine, University of Pennsylvania, Philadelphia, PA, USA
[10]Department of Neurology, Center for Motor Neuron Biology and Disease, Institute for Genomic Medicine, Columbia University, New York, NY, USA
[11]Department of Biological Engineering, Massachusetts Institute of Technology, Cambridge, MA, USA
[12]Department of Neurology, Johns Hopkins School of Medicine, Baltimore, MD, USA
[13]Department of Neurogenetics, Academic Medical Centre, Amsterdam, The Netherlands
[14]Leiden University Medical Center, Leiden, The Netherlands
[15]Department of Medicine, Lung Biology Center, University of California, San Francisco, CA, USA

[16]ALS Multidisciplinary Clinic, Neuromuscular Division, Department of Neurology, Harvard Medical School, Boston, MA, USA
[17]Neurological Clinical Research Institute, Massachusetts General Hospital, Boston, MA, USA
[18]Centre for Neuroscience and Trauma, Blizard Institute, Barts, Queen Mary University of London, London, United Kingdom
[19]The London School of Medicine and Dentistry, Queen Mary University of London, London, United Kingdom
[20]Department of Neurology, Basildon University Hospital, Basildon, United Kingdom
[21]Institute of Neurology, National Hospital for Neurology and Neurosurgery, University College London, London, United Kingdom
[22]The Jackson Laboratory, Bar Harbor, ME, USA
[23]Department of Psychiatry & Human Behavior, School of Medicine, University California, Irvine, CA, USA
[24]Department of Biological Chemistry, School of Medicine, University California, Irvine, CA, USA
[25]Department of Neurobiology and Behavior, School of Biological Sciences, University California, Irvine, CA, USA
[26]Taube/Koret Center for Neurodegenerative Disease Research, Roddenberry Center for Stem Cell Biology and Medicine, Gladstone Institute, San Francisco, CA, USA
[27]Department of Neurology & Sensory Organs, University of Thessaly, Thessaly, Greece
[28]Department of Neurology, Washington University in St. Louis, St. Louis, MO, USA
[29]Centre for Clinical Brain Sciences, Anne Rowling Regenerative Neurology Clinic, University of Edinburgh, Edinburgh, United Kingdom
[30]Euan MacDonald Centre for Motor Neurone Disease Research, University of Edinburgh, Edinburgh, United Kingdom
[31]Department of Molecular Genetics, Weizmann Institute of Science, Rehovot, Israel
[32]Department of Neurology, Icahn School of Medicine at Mount Sinai, New York, NY, USA
[33]Center for Neurodegenerative Disorders, Department of Neurology, the Lewis Katz School of Medicine, Temple University, Philadelphia, PA, USA
[34]Cold Spring Harbor Laboratory, Cold Spring Harbor, NY, USA
[35]Computer Science and Systems Biology Program, Ann Romney Center for Neurological Diseases, Department of Neurology, Brigham and Women’s Hospital, Boston, MA, USA
[36]Division of Genetics in Department of Medicine, Brigham and Women’s Hospital, Harvard Medical School, Boston, MA, USA
[37]Harvard Medical School, Boston, MA, USA
[38]Program in Medical and Population Genetics, Broad Institute, Cambridge, MA, USA
[39]Ann Romney Center for Neurologic Diseases, Brigham and Women's Hospital, Harvard Medical School, Boston, MA, USA
[40]Department of Anesthesiology, Stony Brook University, Stony Brook, NY, USA
[41]Section of Infections of the Nervous System, National Institute of Neurological Disorders and Stroke, NIH, Bethesda, MD, USA

[42]Departments of Translational Neuroscience and Neurology, Barrow Neurological Institute, Phoenix, AZ, USA
[43]St. Joseph's Hospital and Medical Center, Department of Neurobiology, Barrow Neurological Institute, St. Joseph's Hospital and Medical Center, Phoenix, AZ, USA
[44]Department of Neurology, Division of Neuromuscular Medicine, Columbia University, New York, NY, USA
[45]Department of Neuropathology, Academic Medical Center, University of Amsterdam, Amsterdam, The Netherlands
[46]Department of Biology and Veterinary and Biomedical Sciences, The Pennsylvania State University, University Park, PA, USA
[47]New York Stem Cell Foundation, Department of Bioengineering, School of Engineering and Applied Sciences, University of Pennsylvania, Philadelphia, PA, USA
[48]Department of Pathology, Fishberg Department of Neuroscience, Friedman Brain Institute, Icahn School of Medicine at Mount Sinai, New York, NY, USA
[49]Ronald M. Loeb Center for Alzheimer's Disease, Icahn School of Medicine at Mount Sinai, New York, NY, USA
[50]Department of Neurology, Harvard Medical School, Neurological Clinical Research Institute, Massachusetts General Hospital, Boston, MA, USA
[51]Department of Neurology, Hospital for Special Surgery and Weill Cornell Medical Center, New York, NY, USA
[52]Medical Genetics, Atlantic Health System, Morristown Medical Center, Morristown, NJ, USA
[53]Overlook Medical Center, Summit, NJ, USA
[54]Center of Clinical Research, Experimental Surgery and Translational Research, Biomedical Research Foundation of the Academy of Athens (BRFAA), 4 Soranou Efesiou Street, 11527, Athens, Greece
[55]1st Department of Neurology, Eginition Hospital, Medical School, National and Kapodistrian University of Athens, Athens, Greece
[56]Neuromuscular/EMG service and ALS/Motor Neuron Disease Clinic, Hebrew University-Hadassah Medical Center, Jerusalem, Israel
[57]Department of Neurology, Cedars-Sinai Medical Center, Los Angeles, CA, USA
[58]Department of Neuroscience, Icahn School of Medicine at Mount Sinai, New York, NY, USA
[59]Department of Genetics and Genomic Sciences, Icahn School of Medicine at Mount Sinai, New York, NY, USA
[60]Harvard Medical School, Department of Physical Medicine & Rehabilitation, Spaulding Rehabilitation Hospital, Boston, MA, USA
[61]Center for Cellular and Molecular Therapeutics, Children's Hospital of Philadelphia, Philadelphia, PA, USA
[62]Department of Genetics, Perelman School of Medicine, University of Pennsylvania, Philadelphia, PA, USA
[63]Centre for Clinical Brain Sciences, University of Edinburgh, Edinburgh, United Kingdom
[64]Euan MacDonald Centre for Motor Neurone Disease Research, University of Edinburgh, Edinburgh, United Kingdom
[65]Department of Neuropathology, Georgetown Brain Bank, Washington DC, USA

[66]Georgetown Lombardi Comprehensive Cancer Center, Georgetown University Medical Center, Washington DC, USA
[67]Neuroradiology Section, Department of Radiology and Biomedical Imaging, University of California, San Francisco, San Francisco, CA, USA
[68]Neuromuscular Diseases Unit, Department of Neurology, Tel Aviv Sourasky Medical Center, Tel-Aviv, Israel
[69]Sackler Faculty of Medicine, Tel-Aviv University, Tel-Aviv, Israel
[70]Department of Neuroscience, University of California San Diego, La Jolla, CA, USA
[71]Department of Neurology, University of Pennsylvania Perelman School of Medicine, Philadelphia, PA, USA
[72]Department of Neurology, Columbia University Medical Center, New York, NY, USA

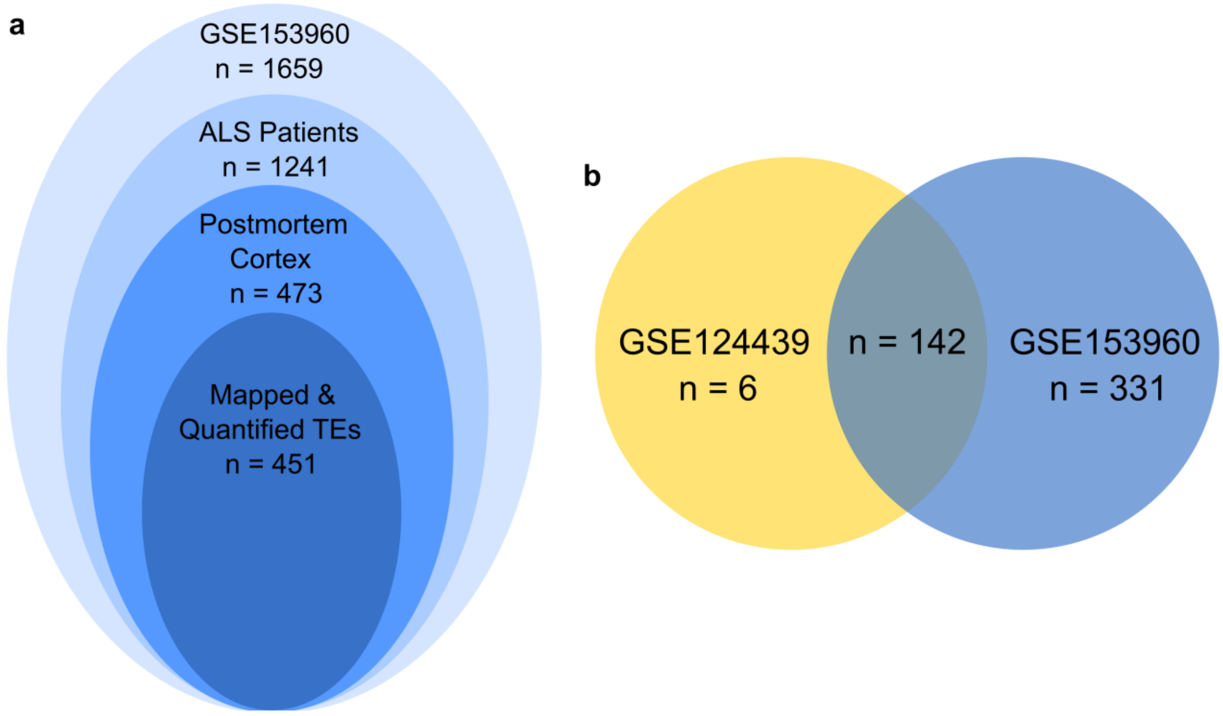

Fig. S1. Overview of the ALS cohort

(**a**) Selection of our ALS cohort from the GSE153960 repository. Transcriptomes associated with the frontal and motor cortex were the only tissue sites considered in this analysis. Control samples are not shown, but included 93 transcriptomes from healthy control donors and 42 from frontotemporal lobar degeneration patients. (**b**) Comparison of common ALS patient postmortem cortex samples between the foundational study from Tam et al. (GSE124439) and the repository utilized in this analysis.

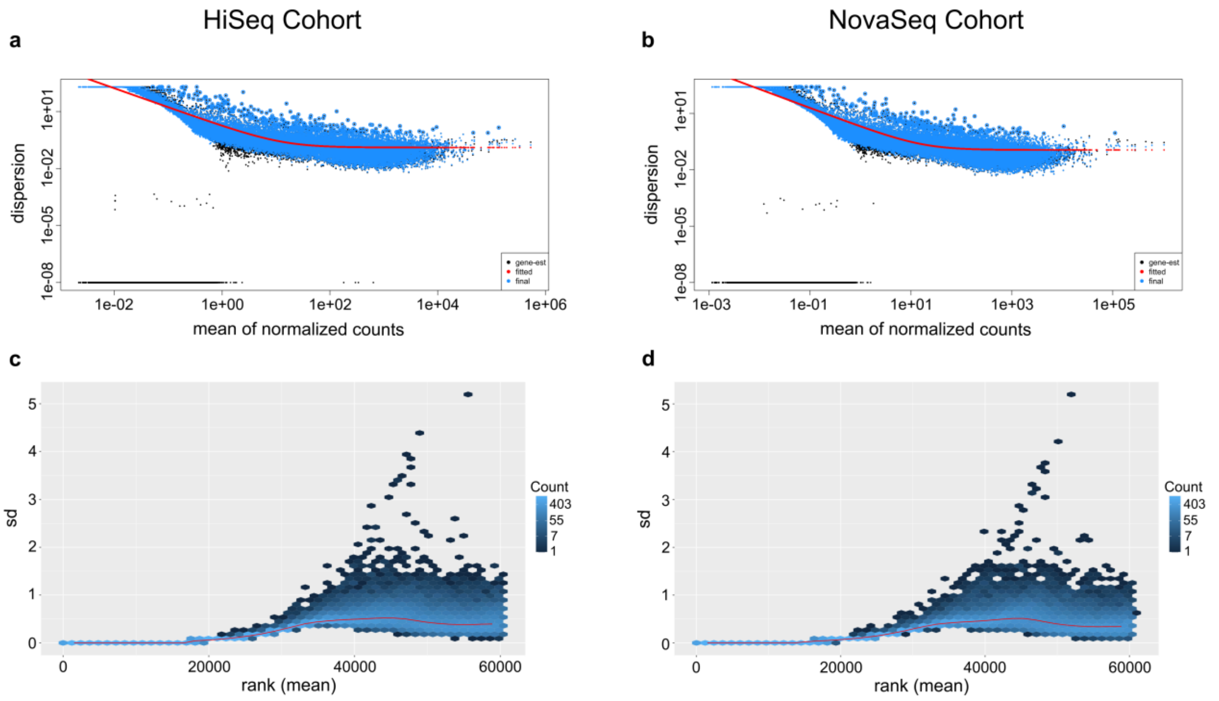

Fig. S2. DESeq2 normalization metrics

(**a**) Dispersion estimation for transcript counts in the HiSeq ALS cohort show gene-wise estimates shrunken towards the fitted estimates. (**b**) Dispersion estimation for transcript counts in the NovaSeq ALS cohort. (**c**) Variance stabilized, mean-standard deviation plot shows roughly the same standard deviation in transcript counts, regardless of gene rank, for the HiSeq ALS cohort. (**d**) Similar standard deviation magnitudes are observed in the variance stabilized, mean-standard deviation plot for the NovaSeq ALS cohort.

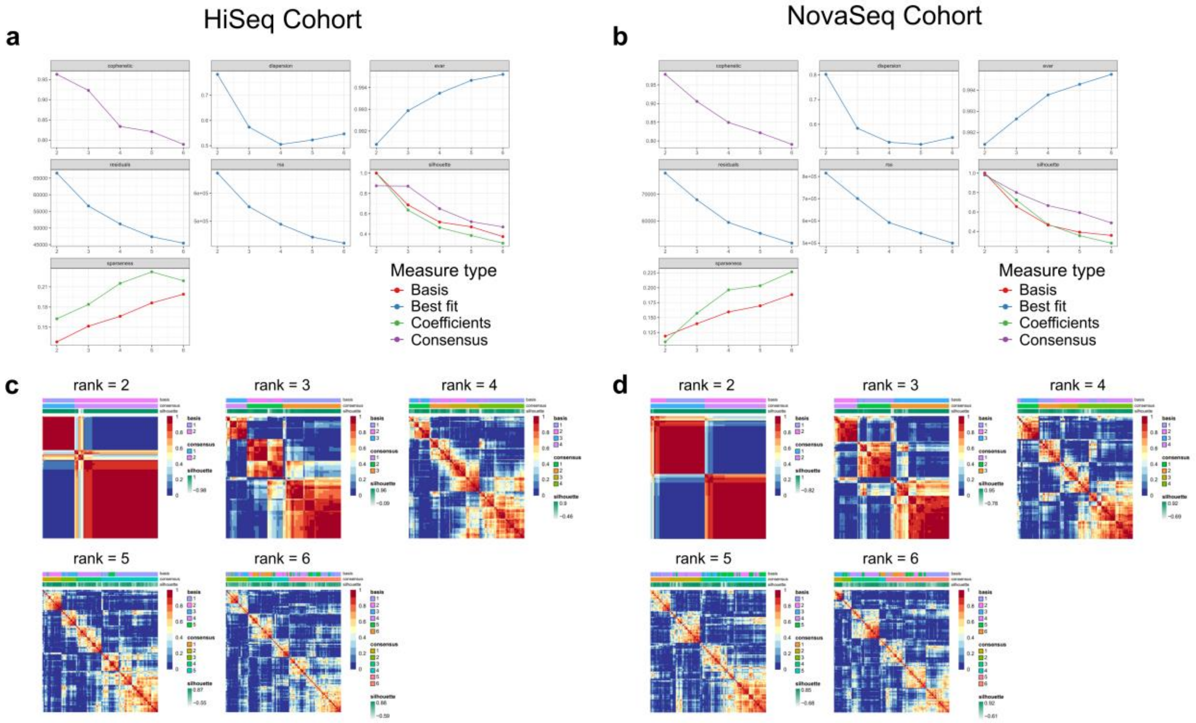

Fig. S3. Estimation of factorization rank

Preliminary unsupervised clustering analysis indicated a factorization rank of 3 was optimal for both the NovaSeq and HiSeq cohorts. (**a**) Non-smooth non-negative matrix factorization metrics for ranks 2-6 in the HiSeq cohort. (**b**) Clustering metrics are shown for the NovaSeq ALS cohort, spanning ranks 2-6. (**c**) Consensus clustering was performed for each rank considered in the HiSeq cohort. (**d**) Consensus clustering results are shown for ranks 2-6 in the NovaSeq cohort.

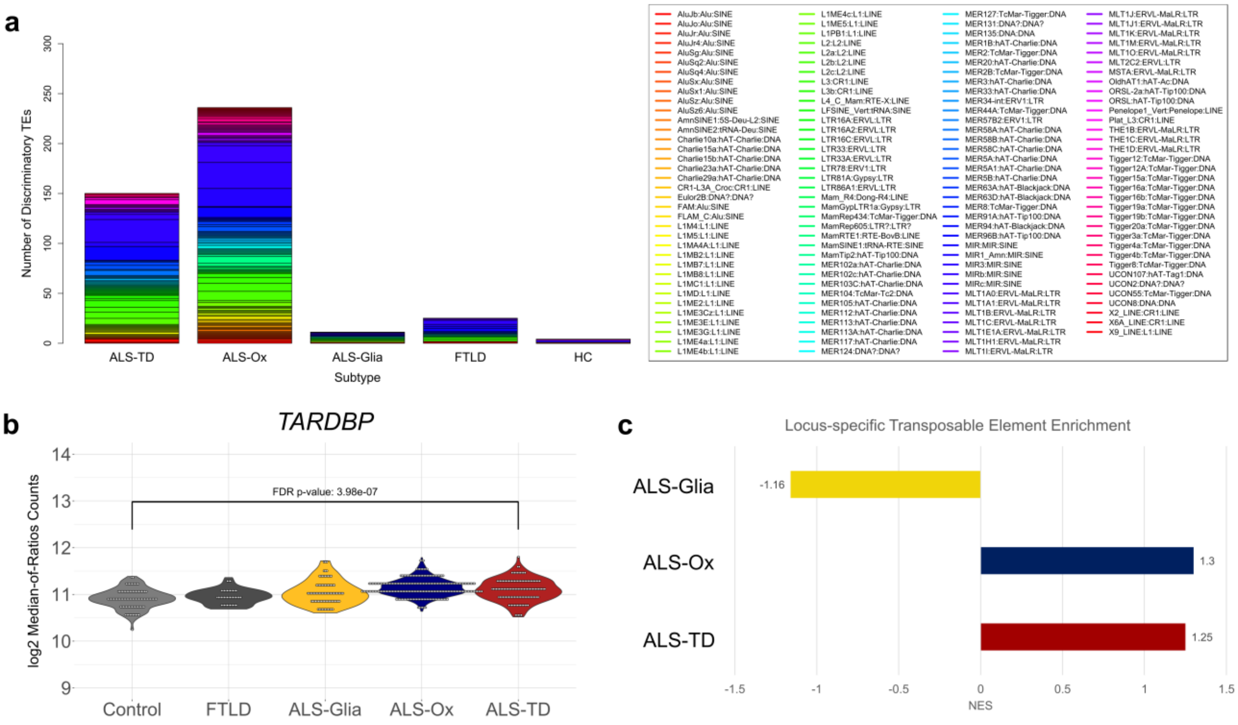

Fig. S4. Consideration of locus-specific transposable elements in ALS spectrum neurodegeneration

(**a**) Locus-specific transposable elements were assigned to the group which demonstrated the largest average expression on the median-of-ratios scale, and reveals characteristic expression in both the TD and Ox subtypes. (**b**) *TARDBP* expression, encoding TDP-43, is shown for healthy controls, patients with frontotemporal degeneration, and each ALS subtype on the DESeq2 median-of-ratios scale. *P*, DESeq2[14] differential expression using the negative binomial distribution, two-tailed, FDR method for multiple hypothesis test correction. Previous works have demonstrated direct interactions between TDP-43 and transposable elements (TEs) and implicated TE subfamilies as subtype specific features[7,99]. However, our results show normalized expression is relatively consistent across ALS subtypes and significant differences in expression are not observed, suggesting *TARDBP* expression is not a defining characteristic of a single subtype. (**c**) A locus-specific transposable element feature set, derived from SQuIRE[13], was utilized to perform TE enrichment using GSEA. Normalized enrichment scores for each of the three subtypes are plotted, with healthy controls specified as the reference. Results indicate that elevated expression of TEs are characteristic of both the ALS-Ox and ALS-TD subtypes.

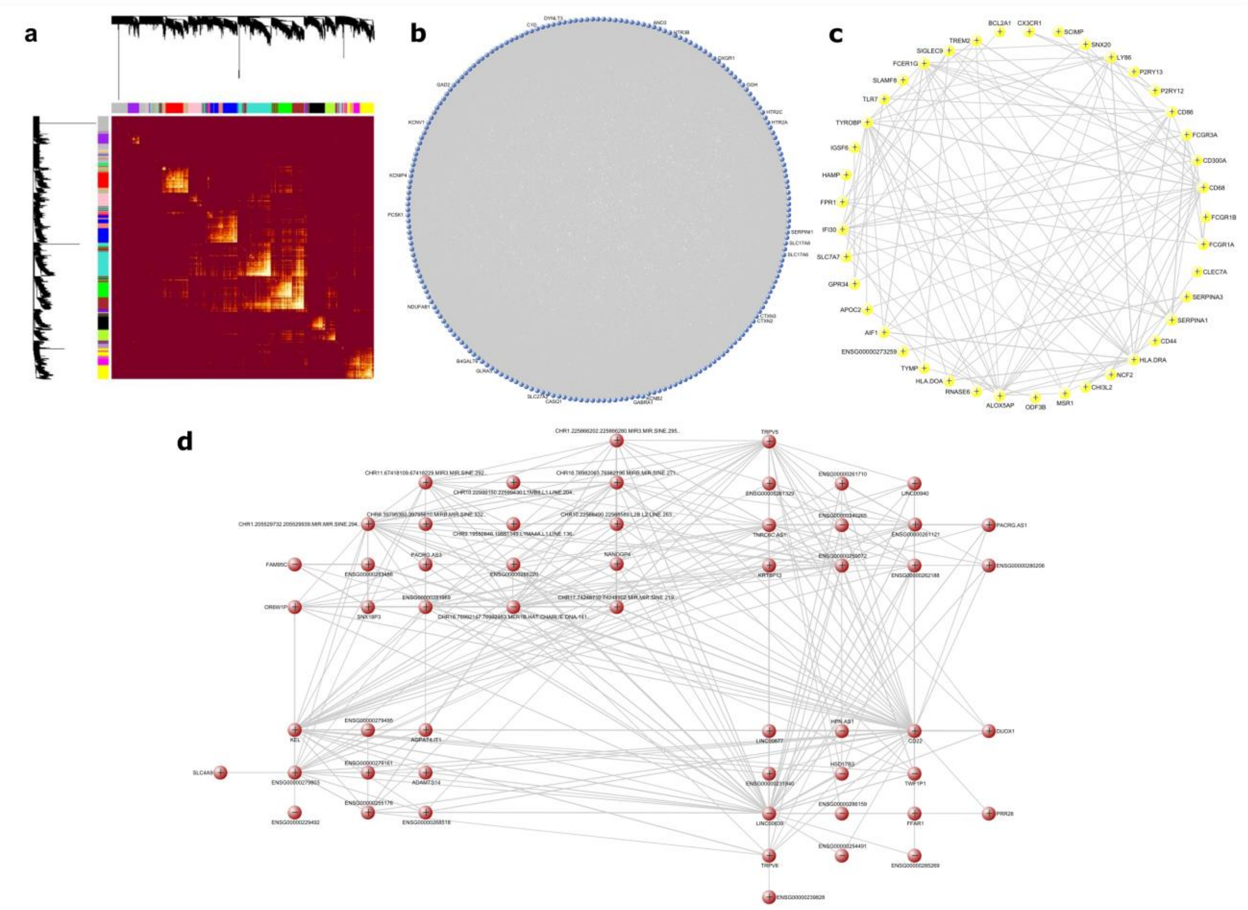

Fig. S5. Eigengene clustering and visualization

(**a**) Eigengene correlation heatmap and clustering dendrogram identifies transcript sets that are co-expressed (eigengenes). The turquoise, purple, and magenta eigengenes were redefined as the navy, gold, and maroon eigengenes, respectively. (**b**) ALS-Ox (navy) eigengene was not significantly enriched for gene ontology, although many features are associated with synaptic signaling and reflect ALS-Ox specific expression (Fig. 6; Fig. S10). (**c**) ALS-Glia (gold) eigengene illustrating immune-related gene expression are positively correlated with ALS age of onset ($p = 0.0008$) and age of death ($p = 0.005$), and negatively correlated with disease duration ($p = 0.0007$, univariate regression between the eigengene and clinical parameter). (**d**) ALS-TD (maroon) eigengene shows long non-coding RNA, pseudogene, and transposable element expression are negatively correlated with ALS age of onset and age of death ($p = 0.03$, univariate regression between the eigengene and clinical parameter).

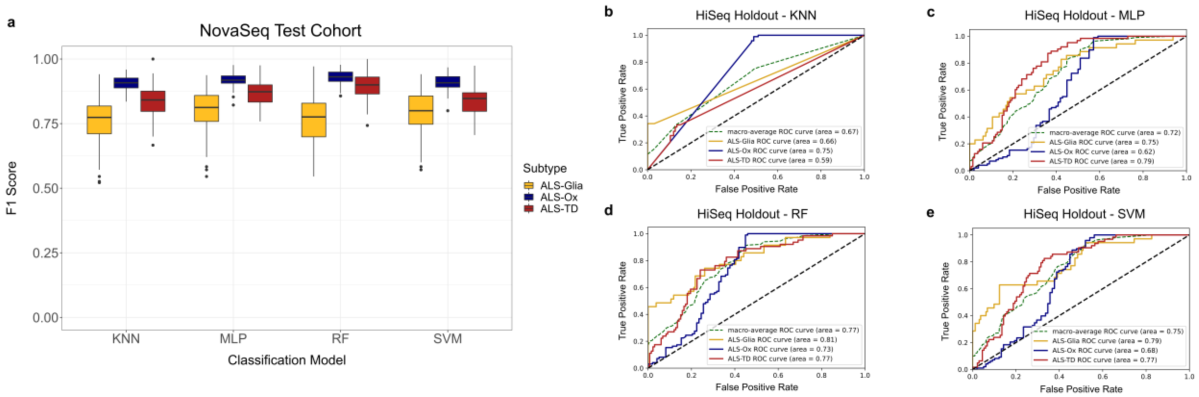

Fig. S6. Performance of four supervised machine learning classifiers in the ALS cohort

(**a**) F1 scores from 100-fold cross validation with the NovaSeq cohort are shown as boxplots, with *n*=208 patients in the training cohort and *n*=89 patients in the test cohort. Four classification methods were considered (KNN, MLP, RF, and linear SVC) and predictive metrics are separated by subtype label. The MLP classifier demonstrated the highest average F1 score for the ALS-Glia subtype (0.80), while the RF classifier showed the best performance when predicting the ALS-Ox (0.93) and ALS-TD subtypes (0.90). The median is indicated by the solid black line, and first and third quartiles are captured by the bounds of the box. Boxplot whiskers are defined as the first and third quartiles –/+ interquartile range times 1.5, respectively, and outliers are denoted as solid black points. Minimum and maximum values are captured by the lowermost and uppermost points, respectively, or whisker bound if no outliers are shown. (**b**) ROC plot showing false positive rate (1-specificity) versus the true positive rate (sensitivity) for the KNN classifier when applied to the holdout (HiSeq) cohort. Given the multi-class nature of this analysis, three classifiers were constructed accounting for each binary case, using a ‘one-versus-rest’ approach. (**c**) ROC plot showing predictive metrics for the MLP classifier. (**d**) Sensitivity and specificity metrics for the random forest classifier when applied to the holdout cohort. (**e**) ROC plot for the linear SVM classifier show similar performance to the RF and MLP models. Using net reclassification improvement and integrated discrimination improvement methodology no single classifier was observed to outperform the others in the case of Glia vs rest. The SVM classifier was determined to outperform all other classifiers for the Ox vs rest case, and both the MLP and SVM classifiers were superior when compared to the RF model in the TD vs rest case.

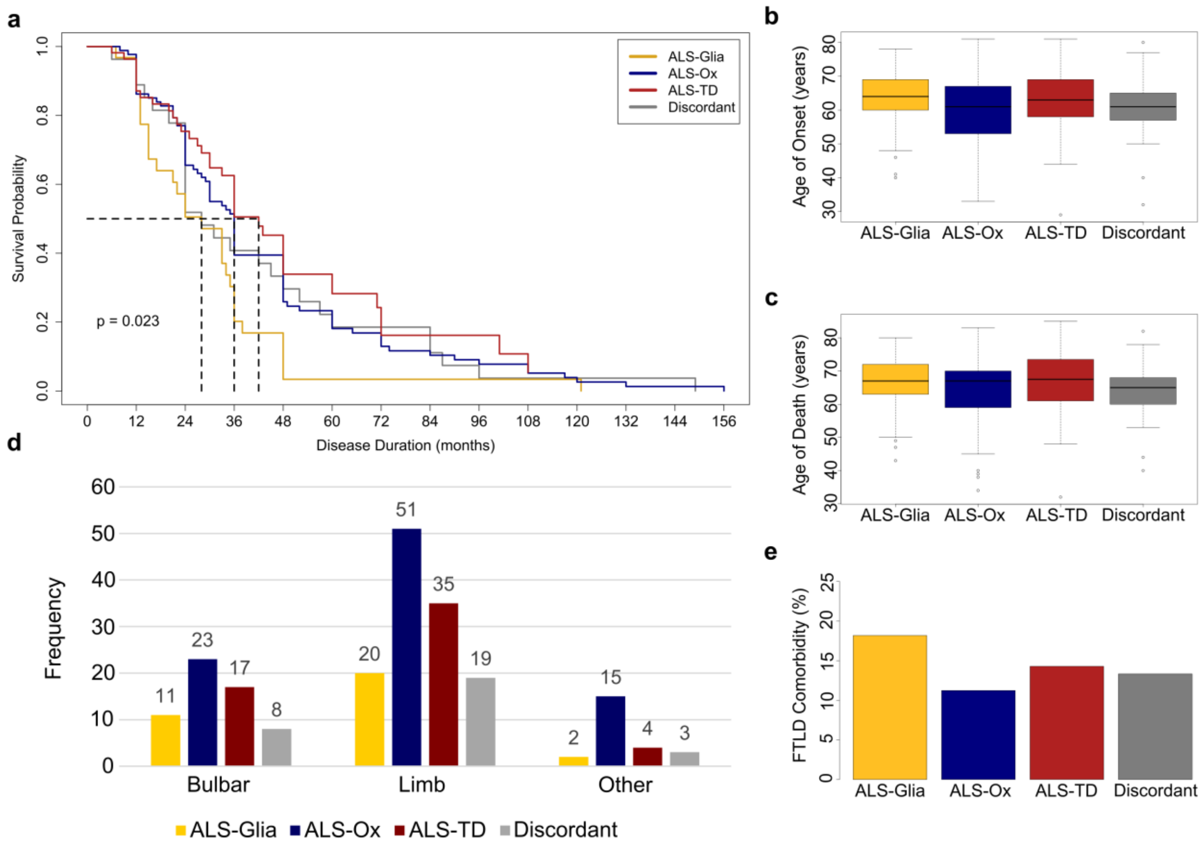

Fig. S7. Inclusion of discordant patients when assessing clinical parameters

(**a**) Kaplan-Meier survival analysis including the three ALS subtypes and 'discordant' patients (*n*=177). Pairwise comparisons showed significant differences in survival between the ALS-Glia and ALS-Ox subtypes (p = 0.015) and ALS-Glia and ALS-TD subtypes (p = 0.0043). *P*, log-rank test. (**b**) Age of ALS symptom onset are plotted as boxplots, separated by disease group (*n*=180). The ALS-Glia subtype shows a nonsignificant trend towards the latest symptom onset. The median is indicated by the solid black line, and first and third quartiles are captured by the bounds of the box. Boxplot whiskers are defined as the first and third quartiles –/+ interquartile range times 1.5, respectively, and outliers are denoted as solid black points. Minimum and maximum values are captured by the lowermost and uppermost points, respectively, or whisker bound if no outliers are shown. (**c**) Age at death are shown for the three ALS subtypes and discordant patients (*n*=208). (**d**) Site of symptom onset are shown for all ALS patients included in this analysis, and a chi-square test of independence suggests site of symptom onset and subtype are not strongly associated. The 'other' category is comprised of axial (4), axial-limb (2), bulbar-limb (4), axial-bulbar (2), generalized (1), and unknown (11) sites of onset. (**e**) Frontotemporal lobar degeneration comorbidity is shown as a percentage, for all ALS patient groups considered in this analysis. A chi-square test of independence again suggests FTLD comorbidity and ALS subtype are not strongly associated.

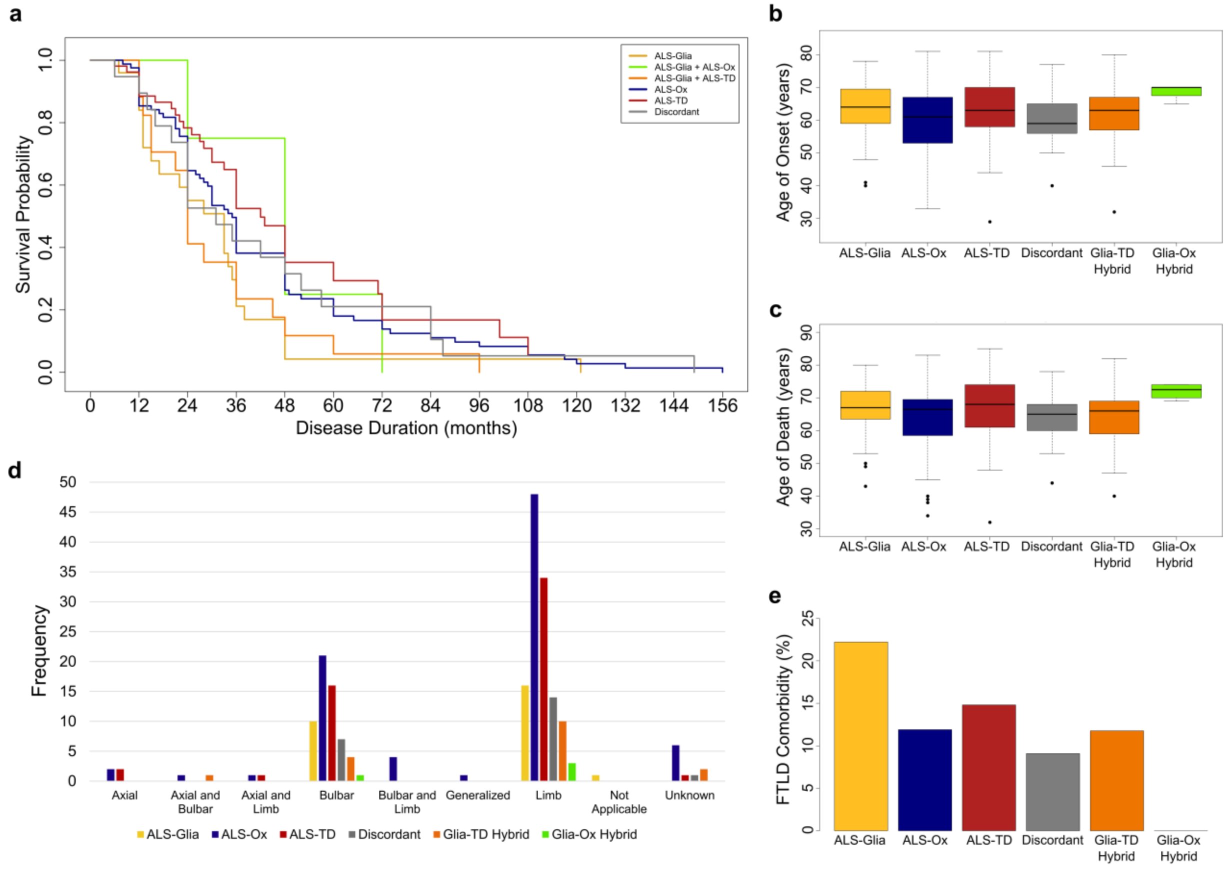

Fig. S8. Clinical parameters in the hybrid subtypes

Patients were assigned to a hybrid subtype if one or more tissue samples passed the thresholds detailed in the 'Methods' section. (**a**) Kaplan-Meier survival analysis including the three ALS subtypes, hybrids, and 'discordant' patients (*n*=177). Interestingly, survival in Glia-TD hybrids mirrors survival in the ALS-Glia subtype, with significant differences observed when compared to the ALS-TD subtype (p = 0.007), and survival differences trending towards significance when compared to the ALS-Ox subtype (p = 0.085). *P*, log-rank test. Our findings suggest the elevated inflammatory phenotype seen in ALS-Glia patients is sufficient to drive fast progression in ALS, irrespective of co-expressed phenotypes, although additional work is needed to assess the consistency of hybrid subtype expression in other cohorts. (**b**) Age of symptom onset, plotted as boxplots, and separated by subtype (*n*=180). No significant differences are observed between the Glia-TD hybrids and other subtypes. The median is indicated by the solid black line, and first and third quartiles are captured by the bounds of the box. Boxplot whiskers are defined as the first and third quartiles –/+ interquartile range times 1.5, respectively, and outliers are denoted as solid black points. Minimum and maximum values are captured by the lowermost and uppermost points, respectively, or whisker bound if no outliers are shown. (**c**) Age of death, separated by subtype (*n*=208). (**d**) Site of symptom onset for all disease subtypes. (**e**) FTLD comorbidity in each disease subtype, presented as a percentage. The small number of Glia-Ox hybrids limits the interpretation of differences observed in survival, age of onset ($p < 0.05$ for all pairwise comparisons), age of death ($p < 0.05$ for all pairwise comparisons), and FTLD comorbidity.

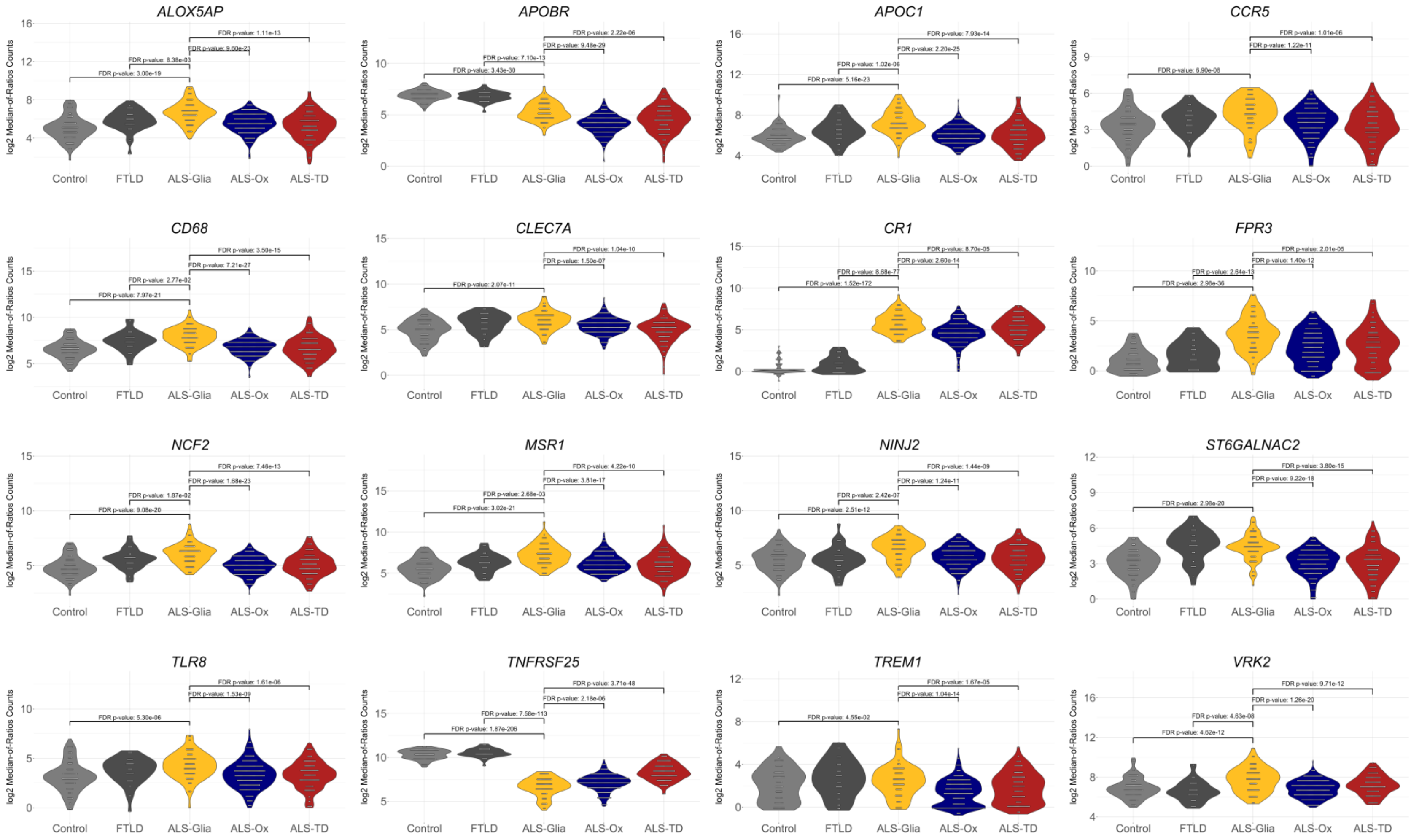

Fig. S9. Supporting features for the ALS-Glia subtype

Violin plots show ALS-Glia specific expression for 16 supporting genes: (*top left) ALOX5AP*, *APOBR*, *APOC1*, *CCR5*, *CD68*, *CLEC7A*, *CR1*, *FPR3*, *MSR1*, *NCF2*, *NINJ2*, *ST6GALNAC2*, *TLR8*, *TNFRSF25*, *TREM1*, and *VRK2*. Genes are generally associated with glial activation, neuroinflammation, and a pro-apoptotic phenotype. *p*-values have been adjusted for RIN, site of collection, and sequencing platform covariates. *P*, DESeq2[14] differential expression using the negative binomial distribution, two-tailed, FDR method for multiple hypothesis test correction.

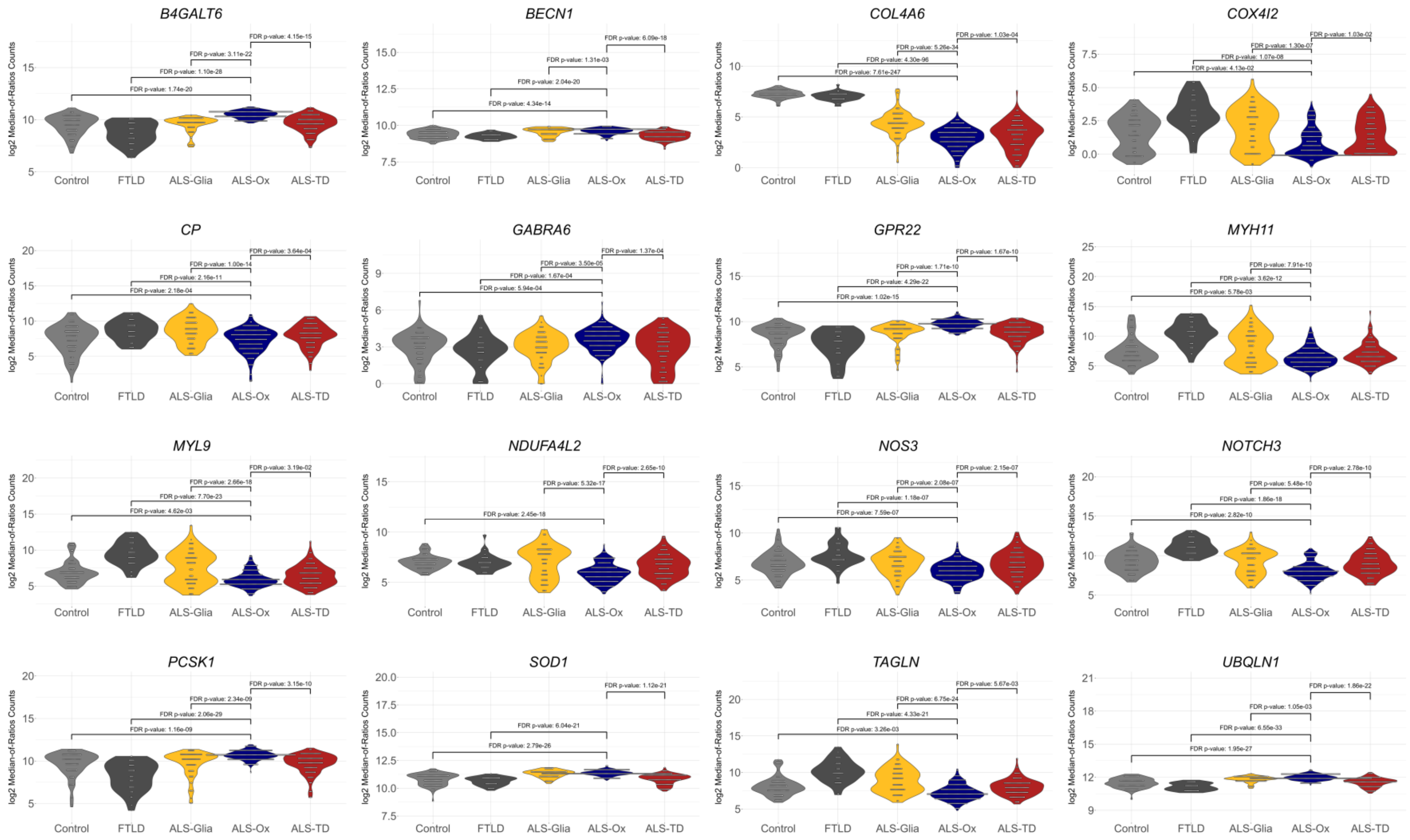

Fig. S10. Supporting features for the ALS-Ox subtype

ALS-Ox specific gene expression is shown as violin plots, and include: (*top left*) *B4GALT6*, *BECN1*, *COL4A6*, *COX4I2*, *CP*, *GABRA6*, *GPR22*, *MYH11*, *MYL9*, *NDUFA4L2*, *NOS3*, *NOTCH3*, *PCSK1*, *SOD1*, *TAGLN*, and *UBQLN1*. Supporting genes are generally associated with synaptic signaling, blood-brain barrier integrity, oxidative stress, and proteotoxic stress. *P*, DESeq2[14] differential expression using the negative binomial distribution, two-tailed, FDR method for multiple hypothesis test correction.

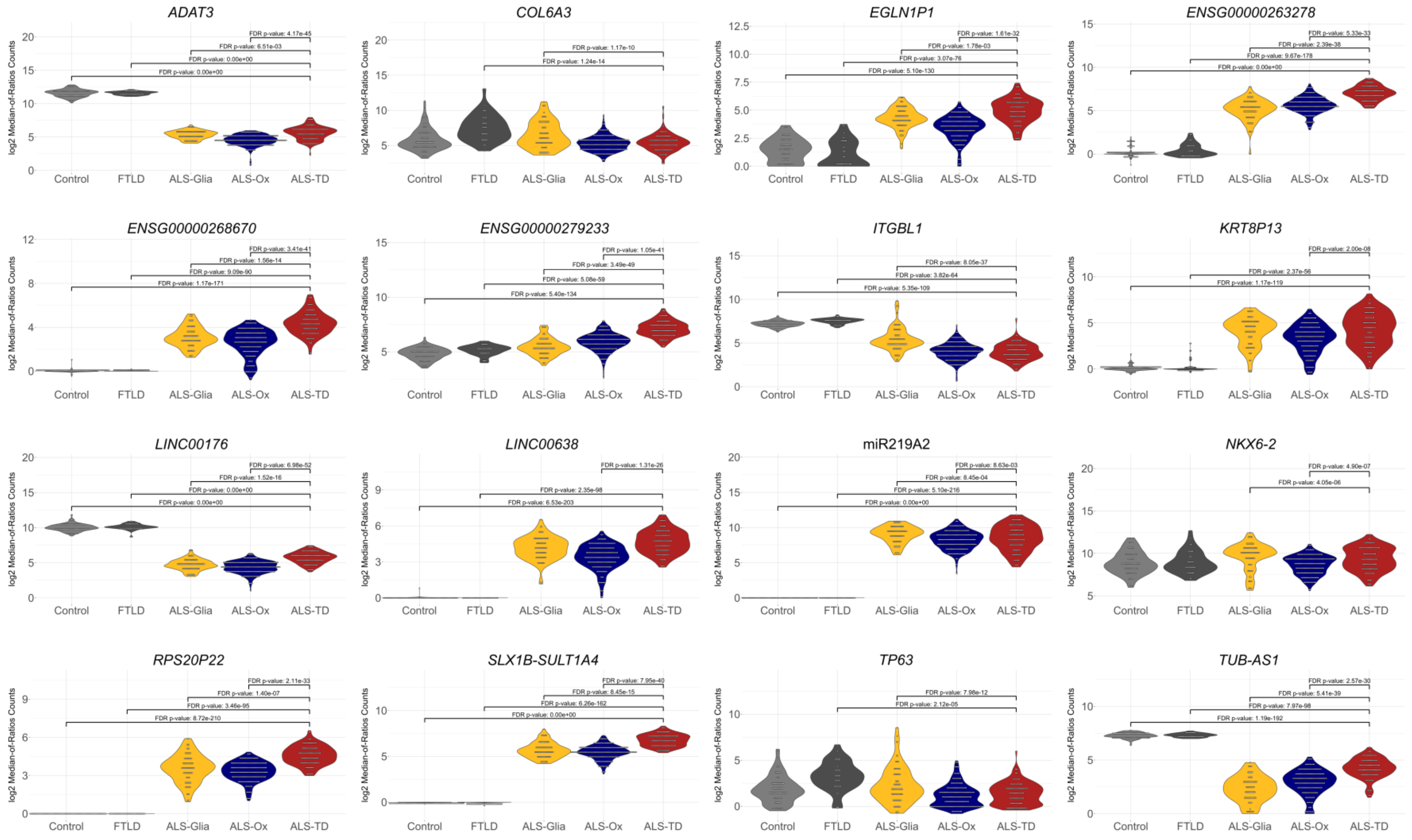

Fig. S11. Supporting features for the ALS-TD subtype

ALS-TD specific feature expression is shown as violin plots, and include: (*top left*) *ADAT3*, *COL6A3*, *EGLN1P1*, *ENSG00000263278*, *ENSG00000268670*, *ENSG00000279233*, *ITGBL1*, *KRT8P13*, *LINC00176*, *LINC00638*, *MIR219A2*, *NKX6-2*, *RPS20P22*, *SLX1B-SULT1A4*, *TP63*, and *TUB-AS1*. Supporting genes are generally associated with transcriptional regulation. *P*, DESeq2[14] differential expression using the negative binomial distribution, two-tailed, FDR method for multiple hypothesis test correction.

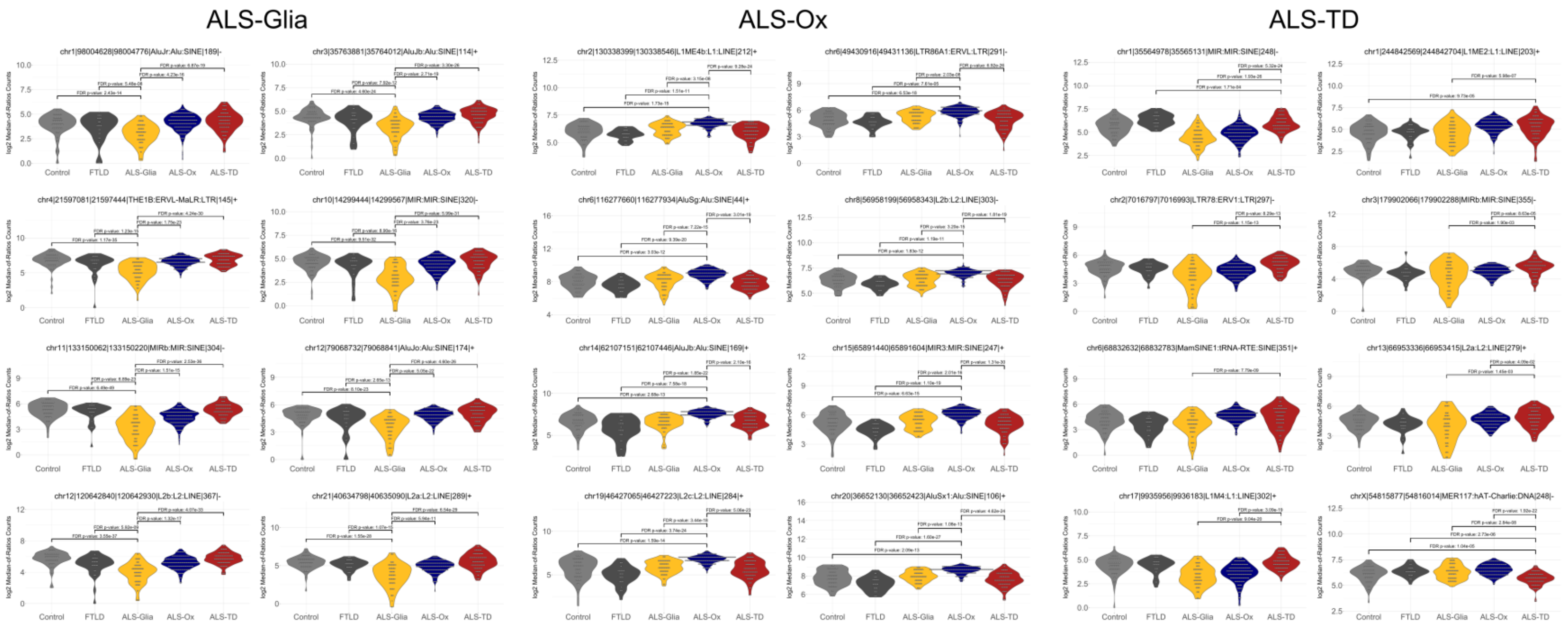

Fig. S12. Subtype-specific transposable element expression
Representative TE features for the ALS-Glia, ALS-Ox, and ALS-TD subtypes. ALS-Ox and ALS-TD subtypes were defined by upregulated expression of long interspersed nuclear elements (LINEs), short interspersed nuclear elements (SINEs), and long terminal repeats (LTRs). The ALS-Glia subtype was defined by downregulated expression of TEs, as compared to other ALS subtypes and controls. *P*, DESeq2[14] differential expression using the negative binomial distribution, two-tailed, FDR method for multiple hypothesis test correction.

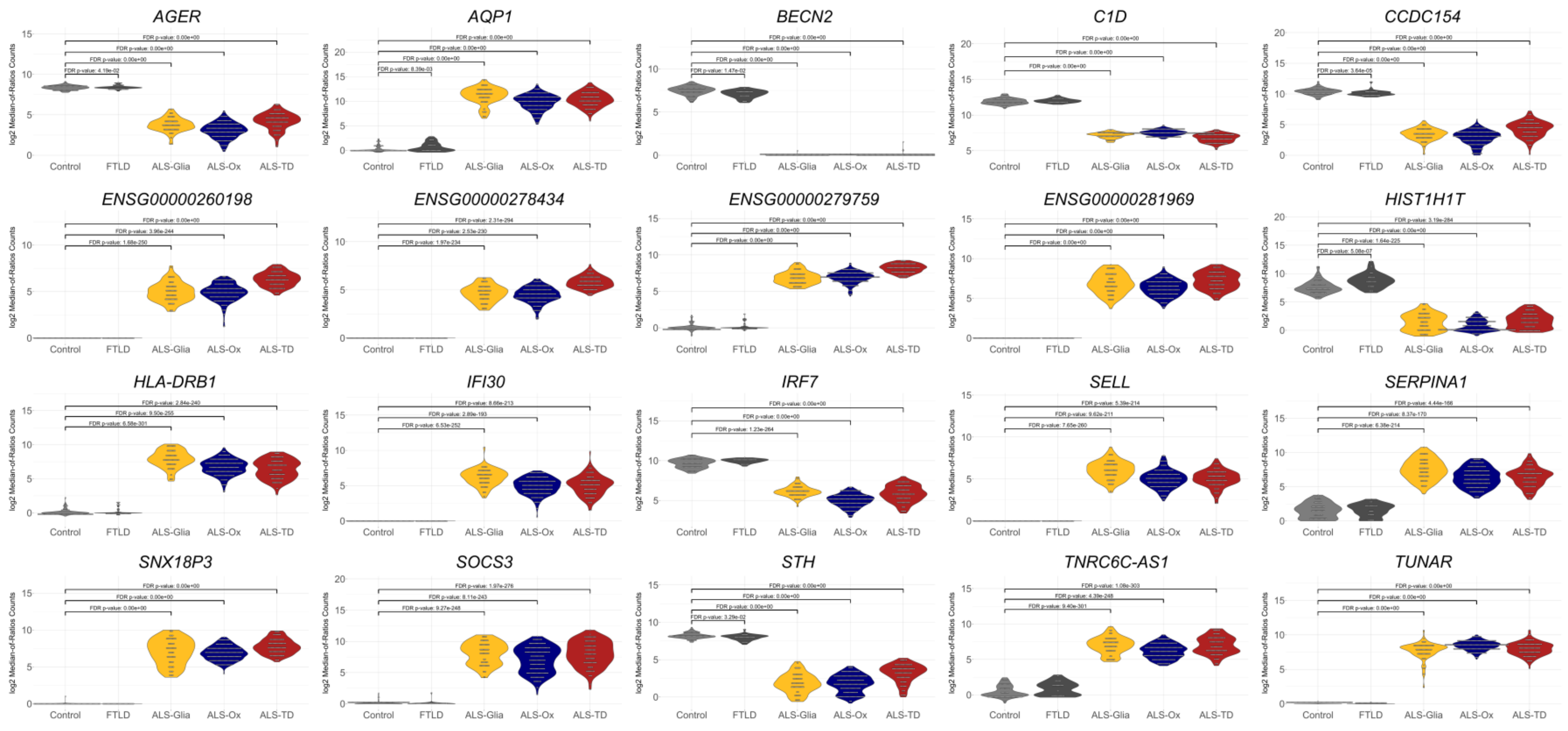

Fig. S13. Characteristic gene expression distinguishes ALS patients from controls

Genes strongly differentially expressed between ALS patients and controls. Violin plots indicate simple thresholding could be utilized to distinguish ALS patients from controls and some genes further show subtype-specific upregulation or downregulation. Of notable interest, elevated expression of *STH* in the brain is known to serve as a marker for Parkinson's and other neurodegenerative diseases, including FTLD, and is observed to be strongly downregulated in all ALS patients. These findings offer a potential marker for the stratification of FTLD patients and ALS patients with FTLD comorbidity. *P*, DESeq2[14] differential expression using the negative binomial distribution, two-tailed, FDR method for multiple hypothesis test correction.

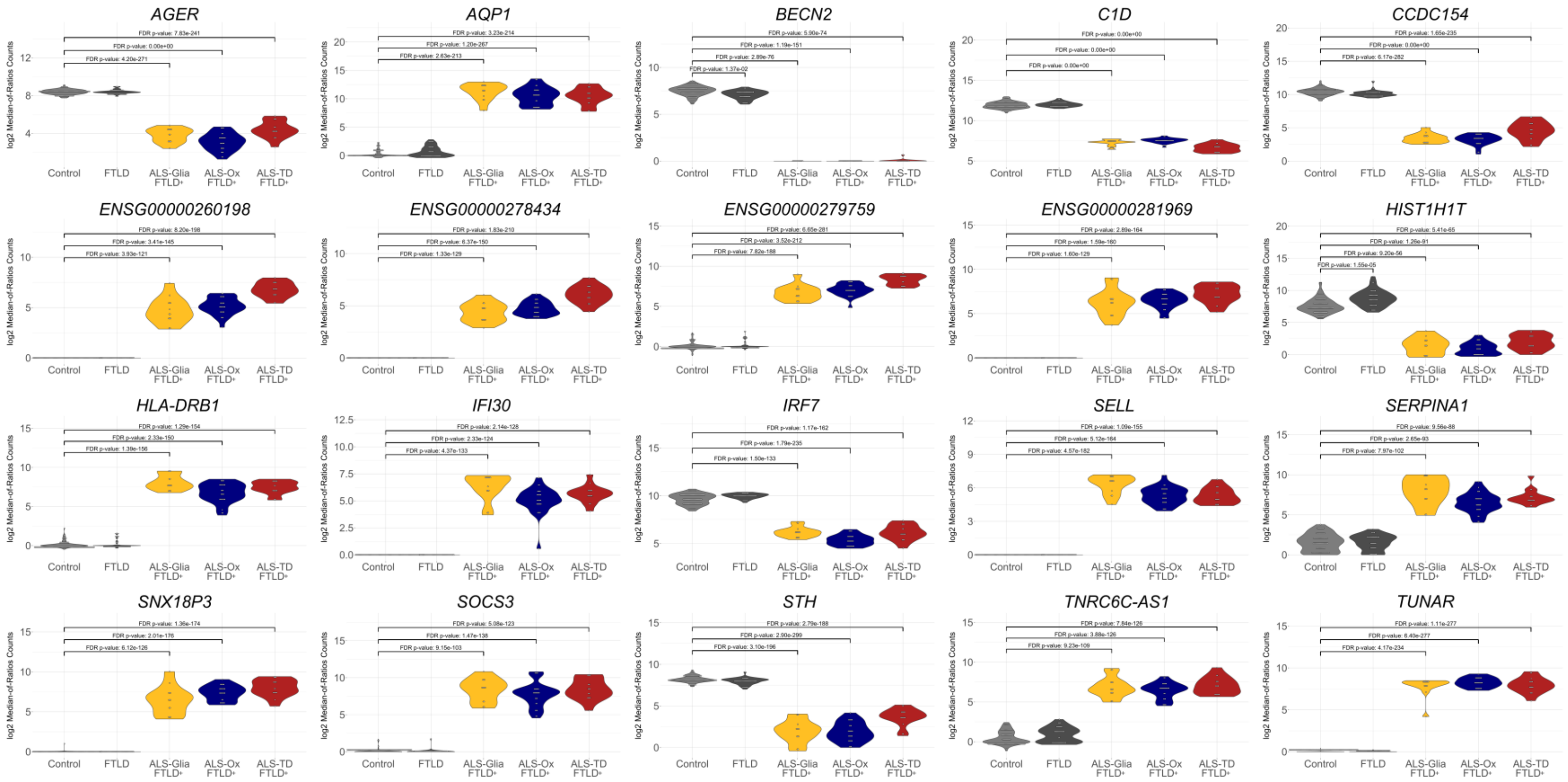

Fig. S14. Univariate analysis considering features distinguishing ALS-FTLD from FTLD
Features presented in Fig. S13 are reconsidered, excluding FTLD– ALS patients. Differential expression between ALS-FTLD and FTLD patients is maintained, further suggesting these features are specific to ALS pathology. *P*, DESeq2[14] differential expression using the negative binomial distribution, two-tailed, FDR method for multiple hypothesis test correction.

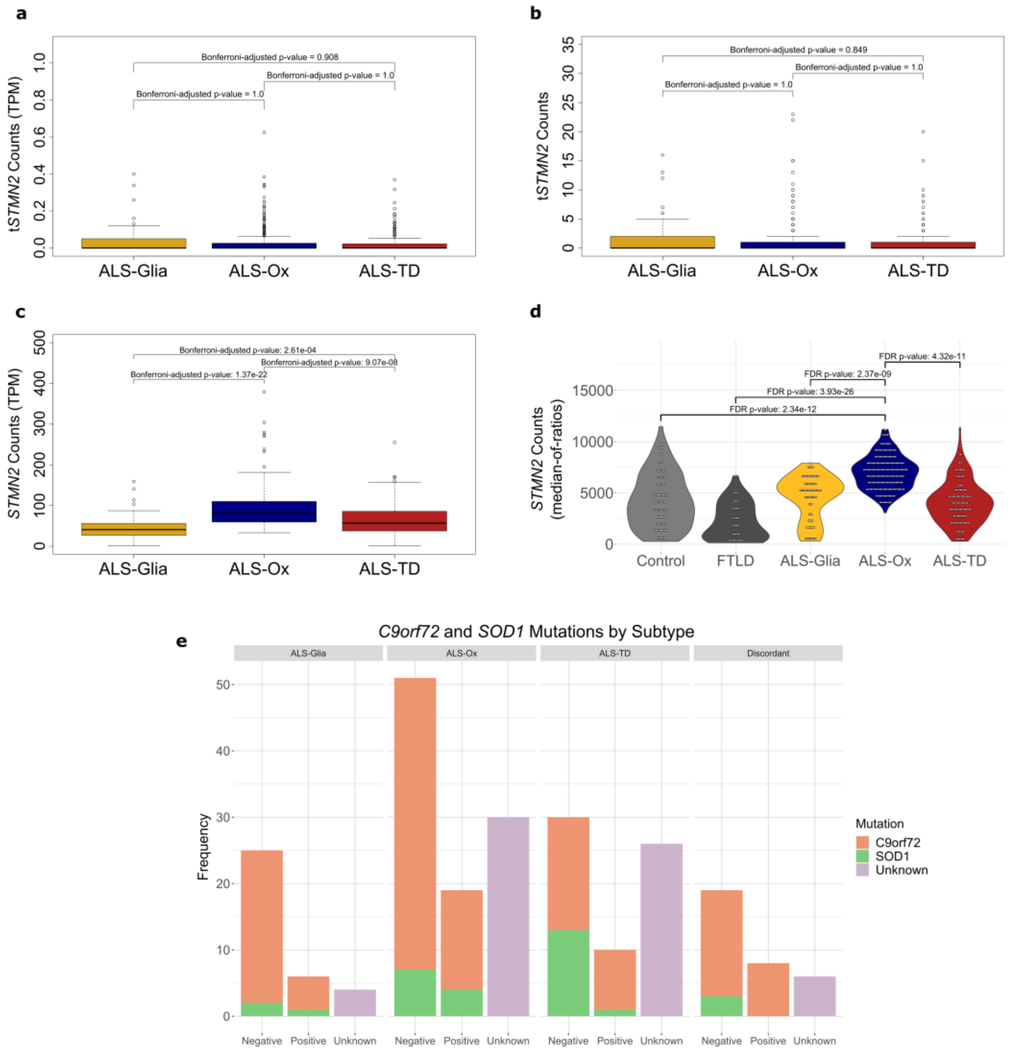

Fig. S15. Truncated and normal length Stathmin-2 in ALS subtypes

The Mann-Whitney U test (two-sided) was used to assess statistical significance in tSTMN-2 expression on both the (**a**) TPM scale and (**b**) raw count scale. After adjusting p-values for multiple hypothesis testing using the Bonferroni method, truncated STMN2 expression was not observed to have any association with ALS subtype. (**c**) Full length transcript STMN2 counts on TPM scale, evaluated using the Mann-Whitney U test (two-sided), with Bonferroni-adjusted p-values shown. (**d**) Full length transcript STMN2 counts on the DESeq2 median-of-ratios scale. Healthy control donors and FTLD patients are included, in an effort to improve the estimation of size factors for normalization. *P*, DESeq2[14] differential expression using the negative binomial distribution, two-tailed, FDR method for multiple hypothesis test correction. (**e**) Stacked bar chart showing *C9orf72* and *SOD1* mutation frequency in the ALS cohort. A chi-squared test of independence was performed to assess mutation dependency on subtype. After removal of the "unknown" categorical variable, the null hypothesis (no association between ALS subtype and common genetic drivers) was accepted for both *C9orf72* (p = 0.47, one-tailed) and *SOD1* (p = 0.21, one-tailed). It is important to note that the limited number of observations for *SOD1* may drive inaccurate estimation of the chi-squared test statistic.

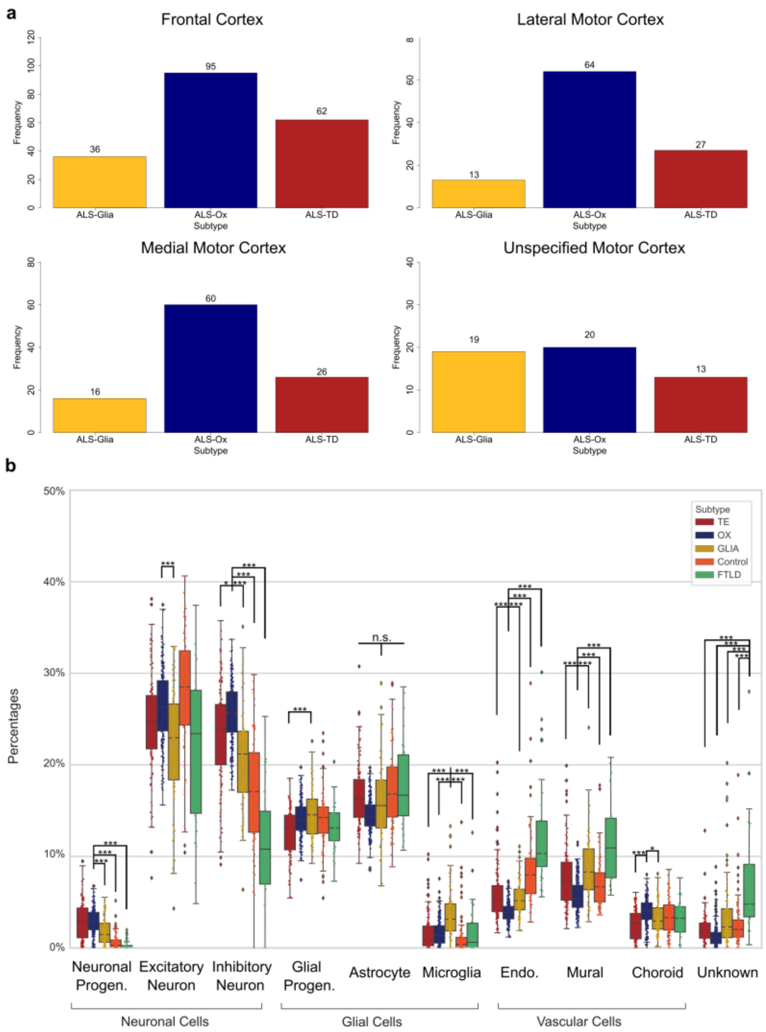

Fig. S16. Cell deconvolution in ALS patients and controls

(**a**) Patient subtypes in each tissue region considered in this study. Approximately the same ratio of Glia, Ox and TD patients are observed in the frontal and specified motor cortices, roughly matching the ratio observed during unsupervised clustering, indicating brain region is not a confounding factor with subtype. (**b**) Cell type percentages in the frontal and motor cortex, considered in the context of ALS patients and controls (*n*=585). Cell deconvolution was performed using CIBERSORT[78], with DESeq2 median-of-ratio counts and references expression from Nowakowski et al.[79]. Significant differences in cell populations was assessed using a Wilcoxon rank sum test (two-sided) with Bonferroni adjustment. Adjusted *p*-values are denoted using the following scheme: *** $p < 0.001$; ** $p < 0.01$; * $p < 0.05$. n.s. – not significant. The median is indicated by the solid black line, and first and third quartiles are captured by the bounds of the box. Boxplot whiskers are defined as the first and third quartiles –/+ interquartile range times 1.5, respectively, and outliers are denoted as solid black points. Minimum and maximum values are captured by the lowermost and uppermost points, respectively, or whisker bound if no outliers are shown. Source data are provided as a Source Data file, including exact *p*-values for all comparisons.

	Cohort Demographics		
A-L: Axial and Limb Onset<br>B-L: Bulbar and Limb Onset<br>A-B: Axial and Bulbar Onset	ALS Spectrum<br>(n = 208)	FTLD<br>(n = 42)	Healthy Control Donors<br>(n = 58)
Sex			
Female	95 (45.7%)	18 (42.9%)	28 (48.3%)
Male	113 (54.3%)	24 (57.1%)	30 (51.7%)
Tissue Site	n = 451	n = 42	n = 93
Frontal Cortex	193 (42.8%)	42 (100%)	56 (60.2%)
Lateral Motor Cortex	104 (23.1%)	0	18 (19.4%)
Medial Motor Cortex	102 (22.6%)	0	19 (20.4%)
Motor Cortex Unspecified	52 (11.5%)	0	0
ALS Subtype		NA	NA
ALS-Glia	33 (15.9%)	–	–
ALS-TD	56 (26.9%)	–	–
ALS-Ox	89 (42.8%)	–	–
ALS-Discordant	30 (14.4%)	–	–
Disease Duration (months)		Not Available	NA
ALS-Glia	29.1 ± 3.81	–	–
ALS-TD	38.1 ± 3.40	–	–
ALS-Ox	41.8 ± 3.18	–	–
ALS-Discordant	42.4 ± 6.35	–	–
Age of Onset (years)		Not Available	NA
ALS-Glia	63.2 ± 1.83	–	–
ALS-TD	62.7 ± 1.68	–	–
ALS-Ox	60.4 ± 1.16	–	–
ALS-Discordant	60.9 ± 1.87	–	–
Site of Onset		NA	NA
ALS-Glia	Bulbar: 11; Limb: 20; Unknown: 2	–	–
ALS-TD	Bulbar: 17; Limb: 35; Axial: 2; A-L: 1; Unknown: 1	–	–
ALS-Ox	Bulbar: 23; Limb: 51; Axial: 2; A-B: 1; A-L: 1; B-L: 4; Generalized: 1; Unknown: 6	–	–
ALS-Discordant	Bulbar: 8; Limb: 19; Unknown: 2; A-B: 1	–	–
Age of Death (years)		66.5 ± 9.5	64.8 ± 15.7 *
ALS-Glia	66.1 ±1.60	–	–
ALS-TD	66.7 ± 1.33	–	–
ALS-Ox	64.0 ± 1.05	–	–
ALS-Discordant	64.0 ± 1.65	–	–
FTLD Comorbidity	27/208 (13.0%)	42/42 (100%)	NA
ALS-Glia	6/33 (18.2%)	–	–
ALS-TD	8/56 (14.3%)	–	–
ALS-Ox	10/89 (11.2%)	–	–
ALS-Discordant	4/30 (13.3%)	–	–

* Three healthy control samples had an age of death listed as "90 or Older". A conservative estimate of 90 years was used for all samples listed as such.

Table S1. Cohort demographics

Cohort demographics for ALS patients, neurological controls, and healthy controls. Disease Duration, Age of Onset, and Age of Death metrics presented as mean ± standard error.

Concordance Matrix		Eshima et al.		
		ALS-TD	ALS-Ox	ALS-Glia
Tam et al.[7]	ALS-TE	21	6	0
	ALS-Ox	9	79	1
	ALS-Glia	1	4	19

Table S2. Subtype concordance matrix
Subtype concordance matrix highlights the strong agreement of subtype labels (85%) between this analysis and the foundational work from Tam et al.[7] for the 140 samples in common.
