# Supplementary material for: Molecular subtypes of ALS are associated with differences in patient prognosis
Source: Nat Commun. 2023 Jan 6;14:95. doi: 10.1038/s41467-022-35494-w (PMC9822908; doi:10.1038/s41467-022-35494-w)
Supplement: Supplementary file 3 — Description of Additional Supplementary Files [file 41467_2022_35494_MOESM3_ESM.pdf]

Description of Additional Supplementary Files

File Name: Supplementary Data 1
Description: Sample IDs and Metadata
GEO IDs and metadata for all postmortem cortex transcriptomes considered in this analysis.

File Name: Supplementary Data 2
Description: Transposable Element Quantification
Transposable element raw counts for all samples considered in this analysis. TE counts reflect the application of the expectation maximization algorithm, to aid in the allocation of multi-mapping reads.

File Name: Supplementary Data 3
Description: Resource, software, and data sources
Resource, software, and data sources for the ALS patient stratification analysis performed in this study.

File Name: Supplementary Data 4
Description: Enrichment feature set
Normalized gene and TE counts for the 1681 features considered during enrichment, network development, and univariate analysis. Feature counts are provided for both the ALS and control cohorts.

File Name: Supplementary Data 5
Description: TE names corresponding to Fig. 2D
Row names for the 426 unique transposable elements presented in Fig. 2E.

File Name: Supplementary Data 6
Description: WGCNA analysis of TEs
Application of WGCNA to a feature set containing transposable elements exclusively. TE eigengenes do not show correlated expression at the subfamily, family or superfamily level – supporting the consideration of locus-specific TEs as unique molecular features.

File Name: Supplementary Data 7
Description: Eigengene membership
Eigengene memberships and gene significances are provided in the context of three clinical parameters: disease duration, age of symptom onset, and age at death.

File Name: Supplementary Data 8
Description: Eigengene enrichment for gene ontology
Enrichment of eigengenes for biological process, molecular function, and cellular component gene ontologies. Bonferroni-adjusted *p*-values are provided and used to assess significant associations between eigengenes and gene ontologies.

File Name: Supplementary Data 9
Description: ALS patient subtype assignment and clinical data
Assignment of ALS patient subtype labels, using the majority agreement approach outlined in Tam et al.[7]. Clinical parameters, such as site of onset, age of onset, age at death, disease duration, fALS genetic characterization, and disease group, are provided for each ALS patient.

File Name: Supplementary Data 10
Description: Differential expression
Pairwise differential expression analysis for ALS subtypes and controls. A multi-factor design equation was utilized for this analysis, accounting for sequencing platform, RIN, site of sample collection, and disease subgroup. A full list of subtype-associated features is also provided and provides the ALS subtype assigned to each feature during clustering with the NovaSeq and HiSeq cohorts.

File Name: Supplementary Data 11
Description: Sample subtype assignment
Robustly assigned subtype labels are provided for all 451 ALS and control patient samples considered in this analysis.

File Name: Supplementary Data 12
Description: Network edge weights for VisANT
WGCNA derived edge weights for eigengene network visualization in VisANT.
