# Supplementary material for: Molecular subtypes of ALS are associated with differences in patient prognosis
Source: Nat Commun. 2023 Jan 6;14:95. doi: 10.1038/s41467-022-35494-w (PMC9822908; doi:10.1038/s41467-022-35494-w)
Supplement: Supplementary file 16 — Reporting Summary [file 41467_2022_35494_MOESM16_ESM.pdf]

nature portfolio

Corresponding author(s): Barbara S. Smith
Last updated by author(s): Nov 8, 2022

Reporting Summary

Nature Portfolio wishes to improve the reproducibility of the work that we publish. This form provides structure for consistency and transparency in reporting. For further information on Nature Portfolio policies, see our Editorial Policies and the Editorial Policy Checklist.

Statistics

For all statistical analyses, confirm that the following items are present in the figure legend, table legend, main text, or Methods section.

n/a	Confirmed	
☐	☒	The exact sample size (*n*) for each experimental group/condition, given as a discrete number and unit of measurement
☐	☒	A statement on whether measurements were taken from distinct samples or whether the same sample was measured repeatedly
☐	☒	The statistical test(s) used AND whether they are one- or two-sided<br>*Only common tests should be described solely by name; describe more complex techniques in the Methods section.*
☐	☒	A description of all covariates tested
☐	☒	A description of any assumptions or corrections, such as tests of normality and adjustment for multiple comparisons
☐	☒	A full description of the statistical parameters including central tendency (e.g. means) or other basic estimates (e.g. regression coefficient) AND variation (e.g. standard deviation) or associated estimates of uncertainty (e.g. confidence intervals)
☐	☒	For null hypothesis testing, the test statistic (e.g. *F*, *t*, *r*) with confidence intervals, effect sizes, degrees of freedom and *P* value noted<br>*Give P values as exact values whenever suitable.*
☒	☐	For Bayesian analysis, information on the choice of priors and Markov chain Monte Carlo settings
☐	☒	For hierarchical and complex designs, identification of the appropriate level for tests and full reporting of outcomes
☒	☐	Estimates of effect sizes (e.g. Cohen's *d*, Pearson's *r*), indicating how they were calculated

Our web collection on statistics for biologists contains articles on many of the points above.

Software and code

Policy information about availability of computer code

Data collection	No software was used for data collection.
Data analysis	SQuIRE (Version 0.9.9.9a-beta) was used for the locus-specific quantification of transposable elements and results have made available as a supplementary data. Code for data preprocessing, unsupervised clustering, WGCNA, bootstrap-based classification, differential expression, and clinical parameter analysis was developed in R (Version 4.0.3, The R Foundation for Statistical Computing, Vienna, Austria) and is publicly available in the Barbara Smith Lab Github repository (https://github.com/BSmithLab/ALSPatientStratification). Code for supervised classification models were developed in Python (Version 3.8.8, Python Software Foundation, Wilmington, DE) and are publicly available in the Barbara Smith Lab and Plaisier Lab Github (https://github.com/plaisier-lab/U5_hNSC_Neural_G0) repositories. Non-negative matrix factorization was implemented using a previously established R package (NMF, Version 0.30.1) and through the SAKE GUI (Version 0.4.0). Differential expression was performed using DESeq2 (Version 1.30.1). Supervised classification models (KNN, MLP, RF, SVC) were developed using the Scikit-Learn framework (Version 0.24.1). Gene enrichment was performed using GSEA (Version 4.1.0) and web-based tool Enrichr. Network visualization was performed in Cytoscape (Version 3.8.2). Gene co-expression was assessed using WGCNA R package (Version 1.70-3). Eigengene visualization was performed using VisANT (Version 5.53). Survival analysis was performed using the survival R package (Version 3.2-10). Cell deconvolution of bulk RNA-seq data was performed using web-based tool CIBERSORTx and R package Seurat (Version 4.0.3).

For manuscripts utilizing custom algorithms or software that are central to the research but not yet described in published literature, software must be made available to editors and reviewers. We strongly encourage code deposition in a community repository (e.g. GitHub). See the Nature Portfolio guidelines for submitting code & software for further information.

Data

Policy information about availability of data

All manuscripts must include a data availability statement. This statement should provide the following information, where applicable:

- Accession codes, unique identifiers, or web links for publicly available datasets
- A description of any restrictions on data availability
- For clinical datasets or third party data, please ensure that the statement adheres to our policy

All raw data files and the RSEM processed gene count matrix utilized in this study are accessible through Gene Expression Omnibus database under accession code GSE153960 [https://www.ncbi.nlm.nih.gov/geo/query/acc.cgi?acc=GSE153960] and NCBI Run Selector database under accession code PRJNA644618 [https://www.ncbi.nlm.nih.gov/Traces/study/?acc=PRJNA644618%20&o=acc_s%3Aa]. Processed data files utilized during this analysis are available as supplemental data or made publicly available at: https://figshare.com/authors/Jarrett_Eshima/13813720.

Human research participants

Policy information about studies involving human research participants and Sex and Gender in Research.

Reporting on sex and gender	Patient sex was assigned using XIST and UTY expression. Roughly an equal number of males and females are maintained in each cohort: ALS (Female: 95 ; Male: 113), FTLD (Female: 18 ; Male: 24), and healthy control donors (Female: 28 ; Male: 30). Genes differentially expressed due to patient sex were removed prior to performing all analyses.
Population characteristics	This study considered frontal and motor postmortem cortex transcriptomes from 451 unique ALS patients, 42 FTLD, and 93 control donors. Patient samples were characterized on two different sequencing platforms, Illumina HiSeq 2500 and NovaSeq 6000, at two separate sites (NYGC and Target ALS). ALS patients were previously assessed for common genetic mutations known to cause ALS, and were found to present with a number of underlying diagnoses including ALS-Alzheimer's, ALS-FTLD, ALS-TDP, and ALS-SOD1.
Recruitment	All frontal and motor postmortem cortex samples from the GSE153960 dataset were initially considered in this analysis.
Ethics oversight	Ethics oversight in postmortem sample collection and RNA-seq analysis was conducted by the New York Genome Center and Target ALS Consortia. All patient data remained de-identified throughout the analysis. Raw RNA-seq patient data is encrypted and stored in a physically secure location.

Note that full information on the approval of the study protocol must also be provided in the manuscript.

Field-specific reporting

Please select the one below that is the best fit for your research. If you are not sure, read the appropriate sections before making your selection.

[x] Life sciences [ ] Behavioural & social sciences [ ] Ecological, evolutionary & environmental sciences

For a reference copy of the document with all sections, see nature.com/documents/nr-reporting-summary-flat.pdf

Life sciences study design

All studies must disclose on these points even when the disclosure is negative.

Sample size	No power analysis was performed to predetermine sample size. ALS sample size (n=208 patients; n=451 transcriptomes) was determined by tissue region (frontal and motor cortex transcriptomes), raw data availability from GSE153960, and successful transposable element quantification using SQuIRE. Within the same data repository, all FTLD and non-neurological control samples (n=135 transcriptomes) corresponding to the frontal and motor cortex were considered, excluding one patient sample which could not be mapped using SQuIRE (n=100 patients).
Data exclusions	Data exclusions are described in the methods section and are summarized in supplemental figure 1 and table S2. In brief, only frontal and motor postmortem cortex samples from GSE153960 were considered in this study. No patients were excluded on the basis of genetic risk. Fewer than 5% of all frontal and motor cortex samples were excluded due to missing paired-end FASTQ files or poor mapping to the transposable element reference genome.
Replication	A table containing ensembl IDs and corresponding gene symbol for the December 2016 Ensembl archive is provided as a supplemental data file to facilitate replication. Patient samples were assigned subtype labels using multiple independent rounds of unsupervised clustering (n=11 rounds) to promote more robust subtype estimation. The bootstrap-based classification strategy (n=1000 iterations) leveraged in this study was performed using a set seed for random number generation, ensuring reproducibility. For the training and testing of supervised classifiers, 100-fold cross validation was applied.

Randomization	No formal randomization method was used. Sex-dependent genes were removed prior to performing all analyses. Differential expression of genes was adjusted for sequencing platform, RIN (scaled), and site of sample collection covariates by inclusion of these variables in the multivariate design equation.
Blinding	Blinding was not relevant to this study, as patient intervention was not performed. All patient samples considered in this study were collected and analyzed postmortem.

Reporting for specific materials, systems and methods

We require information from authors about some types of materials, experimental systems and methods used in many studies. Here, indicate whether each material, system or method listed is relevant to your study. If you are not sure if a list item applies to your research, read the appropriate section before selecting a response.

Materials & experimental systems

n/a	Involved in the study
☒	☐ Antibodies
☒	☐ Eukaryotic cell lines
☒	☐ Palaeontology and archaeology
☒	☐ Animals and other organisms
☒	☐ Clinical data
☒	☐ Dual use research of concern

Methods

n/a	Involved in the study
☒	☐ ChIP-seq
☒	☐ Flow cytometry
☒	☐ MRI-based neuroimaging
